# *KARS*-related diseases: progressive leukoencephalopathy with brainstem and spinal cord calcifications as new phenotype and a review of literature

**DOI:** 10.1186/s13023-018-0788-4

**Published:** 2018-04-04

**Authors:** Anna Ardissone, Davide Tonduti, Andrea Legati, Eleonora Lamantea, Rita Barone, Imen Dorboz, Odile Boespflug-Tanguy, Gabriella Nebbia, Marco Maggioni, Barbara Garavaglia, Isabella Moroni, Laura Farina, Anna Pichiecchio, Simona Orcesi, Luisa Chiapparini, Daniele Ghezzi

**Affiliations:** 1 Child Neurology, Foundation IRCCS Neurological Institute “C. Besta”, Via Celoria 11, 20133 Milan, Italy; 2 Department of Molecular and Translational Medicine DIMET, University of Milan-Bicocca, Milan, Italy; 3 Molecular Neurogenetics, Foundation IRCCS Neurological Institute “C. Besta”, Milan, Italy; 4 Child Neurology and Psychiatry Unit, Department of Clinical and Experimental Medicine, University of Catania, Catania, Italy; 5 INSERM UMR 1141, DHU PROTECT, Paris Diderot University, Sorbonne Paris Cité, France, Paris 06, Paris, France; 6 AP-HP, Department of Neuropediatrics and Metabolic Diseases, National Reference Center for Leukodystrophies, Robert Debré Hospital, Paris, France; 7 Service of Paediatric Hepatology, Department of Paediatrics, Fondazione IRCCS Ca’ Granda Ospedale Maggiore Policlinico, Milan, Italy; 8 Pathology, Fondazione IRCCS Ca’ Granda Ospedale Policlinico Milano, Milan, Italy; 9 Neuroradiology, Foundation IRCCS Neurological Institute “C. Besta”, Milan, Italy; 10 Neuroradiology Department, IRCCS C. Mondino National Neurological Institute, Pavia, Italy; 11 Child Neurology and Psychiatry Unit, C. Mondino National Neurological Institute, Pavia, Italy; 12 Department of Pathophysiology and Transplantation, University of Milan, Milan, Italy

**Keywords:** Mitochondrial disease, KARS, Leukoencephalopathy, Calcifications

## Abstract

**Background:**

*KARS* encodes lysyl- transfer ribonucleic acid (tRNA) synthetase, which catalyzes the aminoacylation of tRNA-Lys in the cytoplasm and mitochondria. Eleven families/sporadic patients and 16 different mutations in *KARS* have been reported to date. The associated clinical phenotype is heterogeneous ranging from early onset encephalopathy to isolated peripheral neuropathy or nonsyndromic hearing impairment. Recently additional presentations including leukoencephalopathy as predominant cerebral involvement or cardiomyopathy, isolated or associated with muscular and cerebral involvement, have been reported. A progressive Leukoencephalopathy with brainstem and spinal cord calcifications was previously described in a singleton patient and in two siblings, without the identification of the genetic cause. We reported here about a new severe phenotype associated with biallelic *KARS* mutations and sharing some common points with the other already reported phenotypes, but with a distinct clinical and neuroimaging picture. Review of *KARS* mutant patients published to date will be also discussed.

**Results:**

Herein, we report the clinical, biochemical and molecular findings of 2 unreported Italian patients affected by developmental delay, acquired microcephaly, spastic tetraparesis, epilepsy, sensory-neural hypoacusia, visual impairment, microcytic hypochromic anaemia and signs of hepatic dysfunction. MRI pattern in our patients was characterized by progressive diffuse leukoencephalopathy and calcifications extending in cerebral, brainstem and cerebellar white matter, with spinal cord involvement. Genetic analysis performed on these 2 patients and in one subject previously described with similar MRI pattern revealed the presence of biallelic mutations in *KARS* in all 3 subjects.

**Conclusions:**

With our report we define the molecular basis of the previously described Leukoencephalopathy with Brainstem and Spinal cord Calcification widening the spectrum of *KARS* related disorders, particularly in childhood onset disease suggestive for mitochondrial impairment. The review of previous cases does not suggest a strict and univocal genotype/phenotype correlation for this highly heterogeneous entity.

Moreover, our cases confirm the usefulness of search for common brain and spine MR imaging pattern and of broad genetic screening, in syndromes clinically resembling mitochondrial disorders in spite of normal biochemical assay.

**Electronic supplementary material:**

The online version of this article (10.1186/s13023-018-0788-4) contains supplementary material, which is available to authorized users.

## Background

*KARS* encodes lysyl- transfer ribonucleic acid (tRNA) synthetase, which catalyzes the aminoacylation of tRNA-Lys in the cytoplasm and mitochondria [1]. Mitochondrial and cytoplasmic aminoacyl–tRNA synthetases (aaRSs) are encoded by distinct nuclear genes, with the exception of KARS and GARS (glycyl–tRNA synthetase) which are present in both cellular compartments [2, 3].

Mutations in *aaRSs* genes have been linked to a growing number of neurological and systemic disorders with heterogeneous phenotype. Eleven families/sporadic patients and 18 different mutations in *KARS* have been reported to date. Phenotype is heterogeneous ranging from early onset encephalopathy [4–7] to isolated peripheral neuropathy [8] or nonsyndromic hearing impairment [9]. Recently, late onset leukoencephalopathy [10] and cardiomyopathy [11, 12] have been reported.

A progressive leukoencephalopathy with brainstem and spinal cord calcifications was previously described as a distinct entity in a singleton patient [13] and in two siblings [14]. We report the clinical, biochemical and molecular findings of 2 unreported patients presenting a similar clinical and radiological picture. Genetic analysis performed on them and in the patient previously described [13] revealed the presence of biallelic mutations in *KARS* in all three subjects. Review of *KARS* mutant patients published to date will also be discussed.

## Methods

All procedures followed were in accordance with the ethical standards of the responsible committee on human experimentation (institutional and national) and with the Helsinki Declaration of 1975, as revised in 2000. Written informed consent was obtained from all individuals or caregivers.

### Genetic analysis

Genomic DNA was extracted from peripheral blood by standard methods. Whole exome sequencing was performed on DNA from patient A, while patient B and C were analyzed using a targeted next generation sequencing (NGS) custom panel containing genes responsible for mitochondrial disorders (Additional file 1). Variants filtering was performed as previously described [15]. Variants identified by WES were validated by Sanger sequencing and resolved on a 3130xl Genetic Analyzer (Applied Biosystems).

### Biochemical studies in skeletal muscle and fibroblasts

Mitochondrial respiratory chain (MRC) activities of complexes I to IV were measured by spectrophotometric methods in supernatants of 800 × g muscle homogenates or in digitonin treated fibroblasts [16]. The activities were normalized to citrate synthase activity, an index of mitochondrial content in the analyzed specimens.

## Results

### Clinical and radiological findings

#### Patient A

Patient A, first child of unrelated Italian parents, showed a progressive leukoencephalopathy with spinal cord calcifications, deafness, hypochromic microcytic anemia and has been already described by Orcesi et al. [13].

#### Patient B

The patient is a 7 year old male. The family history was unremarkable. He was born at term after an uneventful pregnancy. He showed normal psychomotor development until 6 months of age when, a few days after a febrile illness, he had seizures and psychomotor regression. He started phenobarbital treatment. In the following months he showed a slow psychomotor improvement: trunk control was recovered, he was able to walk with support at 12 months, and had been seizures free (he stopped drug at 8 months). At 18 months, after another febrile illness, he presented subacute psychomotor regression and seizures. Valproate treatment was initiated.

He was admitted to our institute at 3 years and 10 months of age. Clinical evaluation showed stunted growth, microcephaly (<3rd percentile), marked scoliosis, nystagmus, poor eye contact and response to sounds, absence of spontaneous movements and postural control, spastic tetraparesis with extrapyramidal signs, absence of language. At last follow up, at 7 years of age, neurological conditions were stable; seizures were not reported.

**Fig. 1:**
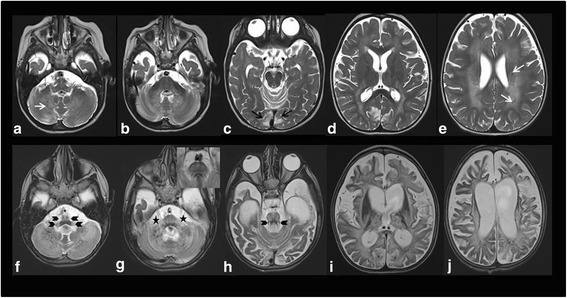
MRI in patient B. TOP, Axial T2WI. performed at 1 year and 9 months revealed diffuse hyperintensity in deep cerebellar white matter, middle cerebellar peduncles, brainstem and bi-hemispheric white matter (**a**, **b**, **c**, **d**). The signal is inhomogeneous for the presence of multiple focal marked hypointensities due to calcifications (**e**, arrows). BOTTOM, Axial T2WI. performed at 3 years and 10 months showed a dramatic extension of the diffuse signal abnormalities in both hemispheres with full involvement of the posterior arm of the internal capsules, external capsules, U fibers and thalami, with relative sparing of the putamina (**h**-**j**). The signal abnormalities extension was also evident in cerebellar white matter, cerebellar peduncles and brainstem (**f**, **g**). Bilateral symmetric hyperintensities in the bulbar pyramids and bulbar lateral regions (arrowheads in **f**), in the superior cerebellar peduncles and in the arciform fibers of their decussation (arrowheads in H) were more evident. The transverse fibers of the pons were prominent and hyperintense (insert in **g**). Both V cranial nerves appeared swollen and hyperintense (stars in **g**), as well as the optic chiasm. Calcarine cortex showed a gliotic hyperintensity (black arrows in **c**). A huge diffuse cerebral atrophy with ventricles and sulci dilatation associated with pronounced cortical thickness was also observed

Abdominal ultrasound- performed before Valproate treatment - disclosed hepatomegaly, echocardiogram was normal. Fundus oculi performed at 6 months disclosed bilateral optic atrophy; since 3 years of age, visual evoked potential showed absence of any responses; brainstem auditory evoked potential suggested profound sensorineural hypoacousia, pure tone audiometry was not performed. Serial EEG revealed poor organization of cerebral activity and multifocal abnormalities. MRI was normal at 6 months. A second MRI (1 year and 9 months) revealed diffuse signal abnormalities in deep cerebellar white matter (WM), middle cerebellar peduncles, brainstem and bi-hemispheric WM (Fig. 1a-e). A third MRI (3 years and 10 months), (Fig. 1h-j) disclosed extension of the diffuse signal abnormalities in bi-hemispheric WM, involving the U fibers (Fig. 1i, j). There was also full involvement of the posterior arm of the internal capsules, external capsules, thalami, cerebellar WM, cerebellar peduncles and brainstem (Fig. 1f-i).T2 shine-through effect on DWI was evident in the areas of the T2 signal abnormalities. Bilateral Calcarine cortex had a malacic appearance, with a gliotic hyperintensity on T2 and focal atrophy. Diffuse cerebral atrophy was also documented (Fig. 1f-i). Spectroscopy showed reduction of NAA and lactate in the centrum semiovale.

**Fig. 2:**
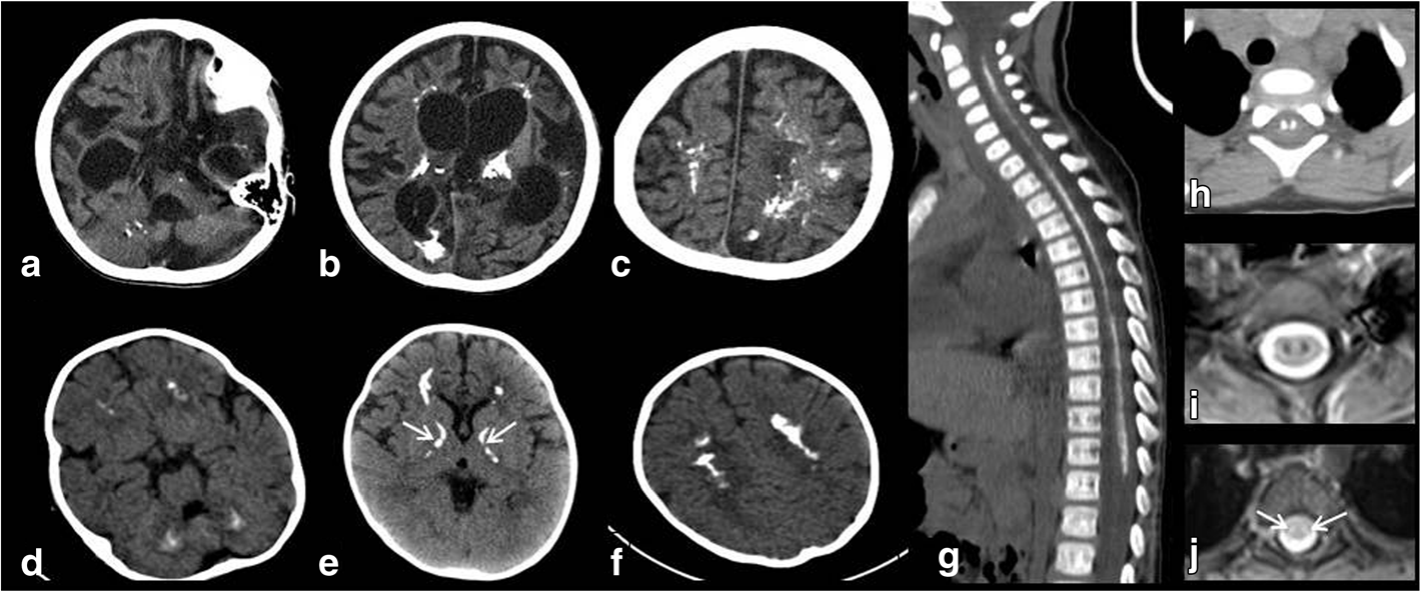
Axial CT images of Patient B (**a**, **b**, **c**) and patient C (**d**, **e**, **f**) during different stages of the diseases, showing “rocks” calcifications in the cerebellar white matter (**a**, **d**), pons (**a**), periventricular white matter, thalami (**b**, **e**), and in the internal capsules, where they have a peculiar «boomerang appearance» (**e**, arrows) and centrum semiovale (**c**, **f**). Spine sagittal (**g**) and axial (**h**) CT of patient A showing extensive «track-like» calcifications along the whole spinal cord, well seen also on axial T2-fast field echo (FFE) MR images at the dorsal level and located in the region of the anterior horns (**i**). Axial T2-FFE MR image of patient C depicts the bilateral hyperintensities on T2WI in the dorsal lateral columns (**j** arrows)

Cranial CT showed calcifications particularly in the periventricular WM, but also evident in the cerebellar WM, pons, thalami, internal capsules and calcarine cortex (Fig. 2a-c). The last brain MRI and CT examinations (7 years) showed worsening of cerebral and cerebellar atrophy and increased cerebral calcifications.

Spinal MRI disclosed on T2WIslight hyperintensity in the lateral columns of both cervical and dorsal spinal cord (Fig. 2j) and spinal CT disclosed calcifications at C6-T1 level.

Blood routine disclosed microcytic hypochromic anemia and mild elevated transaminase levels since first evaluation before valproate treatment. Galactocerebrosidase, arylsulfatases were normal. Plasma lactate and pyruvate levels were elevated: 2872–3042 μmol/l (n.v. 580–2100) and 171 μmol/l (n.v. 5–145) respectively, normal in CSF. Amino acids, creatine and guanidinoacetate were normal. Genetic screening using NGS panel for Aicardi-Goutieres syndrome was negative. A mitochondrial diseases was suspected, muscle biopsy was not possible because of marked hypotrophy. MRC complexes and pyruvate dehydrogenase (PDH) complex activities resulted normal in fibroblasts; analysis of *POLG* was negative.

#### Patient C

This girl was born at term after normal pregnancy and delivery from healthy unrelated parents. At 2 months of age parents started to suspect hearing impairment and at 6 months bilateral cochleopathy was diagnosed on the basis of a type A tympanogram and brainstem auditory evoked potential.

At 12 months she came to our attention. Neurological evaluation showed spastic tetraplegia and microcephaly; she was able to fixate and track a visual target, but was not able to reach an object; spontaneous movements were poor and non-finalized, postural controls absent. At last follow up, at 18 months of age, neurological conditions were stable.

Cerebral MRI (at 6, 12 and 18 months) showed progressive bilateral diffuse supra e infra-tentorial WM abnormalities with involvement of centrum semiovale, corona radiata, initially sparing U fibers; there was also involvement of the posterior arm of the internal capsules, external capsules, thalami, cerebellar and deep WM and brainstem, while putamina were relatively spared. In particular, in the brainstem we noticed bilateral symmetric hyperintensities in the bulbar pyramids and lateral regions, involvement of the superior cerebellar peduncles and in the arciform fibers of their decussation at the level of the mesencephalon. Both V cranial nerves appeared slightly hyperintense on T2WI. Progressive cerebral calcifications were also evident on CT (Fig. 2d-f), initially involving internal capsules, deep and periventricular WM (at 6 months) and since 12 months dentate nuclei.

Spinal MRI performed at 12 months documented bilateral hyperintensities on T2WI in the cervical and dorsal lateral columns (Fig. 2j) and CT showed calcifications at 12 and 18 months.

Visual evoked potentials was slightly delayed at 12 months and severely abnormal at 18, electroretinogram was normal, fundus oculi showed diffuse mild depigmentation; peripheral nerves conductions were normal; EEG showed poor organization of background activity with fronto-centro-temporal spikes at 12 months, increased at 18 months.

Biochemical exams revealed microcytic hypochromic anaemia with normal iron concentration, high level of plasma lactate (3312 μmol/l) and pyruvate (199 μmol/l) with normal CSF concentration. MRC complexes activities resulted normal in fibroblasts.

**Fig. 3:**
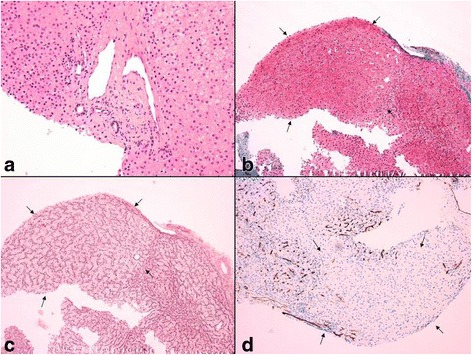
Histological liver changes in patient C: mild sclerotic portal space with irregular venous vessels (**a**, hematoxylin and eosin stain, 200×); vaguely nodular lobular area with compressed peripheral trabeculae (arrows) can be highlighted by Masson’s trichrome (**b**, 100×) and Reticulin (**c**, 100×) stainings, and CD34 immunohistochemistry (**d**, 100×)

Abdominal ultrasound revealed inhomogeneous echogenicity, with nodular aspects, liver function tests showed slight elevation of serum transaminases, with normal synthetic function and no signs of cholestasis. Liver biopsy showed mild portal sclerosis with mild distortion of venous portal vessels, without any significant inflammatory infiltrate; lobular parenchyma demonstrated focal enlarged trabeculae with compression of the peripheral hepatocytes, and preserved reticular network without fibrosis. These findings were suggestive of a vascular disturbance with nodular regenerative hyperplasia (Fig. 3).

### Genetic studies

Whole exome sequencing was performed on patient A; following a filtering strategy that enriched for rare (MAF of < 1%) nonsynonymous/splice variants that segregated in a recessive manner, a short list of candidate genes was obtained but without any known gene associated with WM disease (Additional file 2). Independently, NGS-based screening of genes associated with mitochondrial disorders performed on patient B disclosed two heterozygous variants in *KARS (*NM_001130089.1)*:* c.1124A > G/p.Tyr375Cys and c.381C > G/p.Phe127Leu. These variants and their segregation in the family were confirmed by Sanger sequencing.

Given the striking similarities of clinical and neuroimaging features between patient B and patient A, WES data from the latter were reconsidered and a likely causative role was assigned to the homozygous variant c.1514G > A/p.Arg505His in *KARS*. Similarly, because of her clinical presentation an NGS-based screening of *KARS* was performed on patient C, leading to the identification of two heterozygous variants: c.815 T > G/p.Phe272Cys and c.1043G > A/p.Arg348His. All the identified *KARS* variants had an extremely low frequency in public databases (< 0.01%), are predicted to be deleterious and hit phylogenetically conserved amino acid residues, supporting their causative role (Additional file 3). Notably, the c.1514G > A/p.Arg505His found in patient A has been recently reported in compound heterozygosity with another missense change in two siblings with early-onset hearing loss and leukoencephalopathy and its pathogenicity has been experimentally proven [10].

## Discussion

Mutations in different *aaRSs* have been associated with an increasing number of phenotypes [2, 3]. Encephalopathy is the most common phenotype, but other extra neurological symptoms have been reported: sideroblastic anemia (YARS2 [17, 18]), cardiomyopathy and myopathy (YARS2 [19], GARS [20], KARS [11, 12], tubulopathy (SARS2 [21]), ovarian failure (AARS2 [22], HARS2 [23], LARS2 [24]), hepatopathy (FARS2 [25], EARS2 [26]) and hearing loss (HARS2 [23], LARS2 [25], KARS [9]).

Up to now, 11 families/sporadic patients and 18 mutations in *KARS* have been reported (Tables 1 and 2).

**Table 1:** Clinical, instrumental, biochemical and molecular findings in published patients with *KARS* mutations

Patient	Family	Age onset	Symptoms at onset	Clinical finding	Brain MRI	Visceral involvement/ systemic symptoms	Age at last follow up	Reference
Pt 1	Fam. 1	Adulthood	Intermediate CMT, developmental delay, self-abusive behavior, dysmorphic features, and vestibular Schwannoma		n.a.	n.a.	n.a.	McLaughlin HM et al. 2010 [8]
Pts 2–8	Fam. 2	Childhood	Nonsyndromic hearing impairment		n.a.	n.a.	n.a.	Santos-Cortez RL et al. 2013 [9]
Pts 9–14	Fam. 3–4	Childhood	Nonsyndromic hearing impairment		n.a.	n.a.	n.a.	Santos-Cortez RL et al. 2013 [9]
Pt 15	Fam. 5	16 months	n.a.	Psychomotor delay, hearing loss, strabismus, ophthalmoplegia, dystonia	Normal (6 months)	n.a.	Died at 3 years	Lieber et al. 2013 [4]
Pt 16	Fam. 6	6 weeks	Visual impairment	Microcephaly, psychomotor delay, seizures	Symmetrical thinning of cerebral WM and the corpus callosum (9 months); progression of myelination, symmetrical deep WM abnormalities (20 months)	n.a.	10 years	McMillan et al. 2015 [5]
Pt 17		5 weeks			Thin cc (4 months); loss of subcortical white matter volume, deep sulcation, and hypogenesis cc (5.3 years)	n.a.	5 years	McMillan et al. 2015 [5]
Pt 18	Fam. 7	72 days	Nystagmus, failure to thrive, inability to fixate, microcephaly, hypertonicity, and extreme irritability	Microcephaly and failure to thrive, psychomotor delay, seizures (West syndrome)	Normal (age not reported)	n.a.	18 months	Joshi et al. 2016 [6]
Pt 19	Fam. 8	9 months	n.a.	Developmental delay, seizures, nystagmus	n.a.	Hypertrophic cardiomyopathy	n.a.	Kohda et al. 2016 [11]
Pt 20	Fam. 9	18 months	Mild psychomotor delay	Mild myopathy,mild intellectual disability	Normal (13 years)	Hypertrophic cardiomyopathy	14 years	Verrigni et al. 2016 [12]
Pts 21–22	Fam. 10	First years	Hearing loss	Hearing loss, cognitive and psychiatric symptoms	Symmetrical confluent abnormalities in the frontal, periventricular WM and in the cc	no	26–21 years	Zhou et al. 2017 [10]
Pt 23	Fam. 11	First months	Developmental delay, microcephaly	Global developmental delay, microcephaly, hypotonia, seizures and sensorineural hearing loss	n.a. (CT: calcification)	n.a.	18 years	Murray et al. 2017 [7]
Pt 24		First months	Hearing loss		n.a.	n.a.	15 years	
Pt 25	Fam. 12	3 months	Hypoacousia	Severe psychomotor delay, microcephaly, visual impairment, spastic tetraparesis	Progressive Leukoencephalopathy with Brainstem and Spinal cord Calcifications (see text for detail)	Microcytic hypochromic anemia	19 months	This report (Pt A); Orcesi et al. 2011 [13]
Pt 26	Fam. 13	6 months	Seizures and psychomotor regression	Severe psychomotor delay, microcephaly, visual impairment, spastic tetraparesis with extrapyramidal signs,scoliosis	Progressive Leukoencephalopathy with Brainstem and Spinal cord Calcifications (see text for detail)	Hepatomegaly/microcytic hypochromic anemia	7 years	This report (Pt B)
Pt 27	Fam. 14	6 months	hypoacousia	Psychomotor delay, microcephaly, visual impairment, spastic tetraplegia	Progressive Leukoencephalopathy with Brainstem and Spinal cord Calcifications (see text for detail)	Hepatopathy/microcytic hypochromic anaemia	18 months	This report (Pt C)

**Table 2:** biochemical and molecular findings in published patients with KARS mutations

Patient	Family	Nucleotide mutations	Protein change(s)	Lactate	MRC activity	Reference
Pt 1	Fam. 1	c.398 T > A; c.514_515insTT	p.Leu133His; p.Tyr173Serfs*9	n.a.	n.a.	McLaughlin HM et al. 2010 [8]
Pts 2–8	Fam. 2	c.1129G > A; c.1129G > A	p.Asp377Asn	n.a.	n.a.	Santos-Cortez RL et al. 2013 [9]
Pts 9–14	Fam. 3–4	c.517 T > C; c.517 T > C	p.Tyr173His	n.a.	n.a.	Santos-Cortez RL et al. 2013 [9]
Pt 15	Fam. 5	c.1760C > T; c.683C > T	p.Thr587Met; p.Pro228Leu	Elevated (CSF)	n.a.	Lieber et al. 2013 [4]
Pt 16	Fam. 6	c.1396C > T; c.1657G > A	p.Arg466Trp; p.Glu553Lys	Normal (plasma)	n.a.	McMillan et al. 2015 [5]
Pt 17				n.a.	n.a.	McMillan et al. 2015 [5]
Pt 18	Fam. 7	c.169G > C; deletion at chr16:75672800–75,680,400	p.Ala57Pro; loss of starting codon	n.a.	n.a.	Joshi et al. 2016 [6]
Pt 19	Fam. 8	c.1037 T > C; c.1427 T > A	p.Ile346Thr; p.Val476Asp	Elevated	cI-IV defects (fibroblast)	Kohda et al. 2016 [11]
Pt 20	Fam. 9	c.1133 T > A; c.1253C > G	p.Leu378His; p.Pro418Arg	Elevated	cI-IV defects (muscle)	Verrigni et al. 2016 [12]
Pts 21–22	Fam. 10	c.1514G > A; c.1597C > T	p.Arg505His; p.Pro533Ser	n.a.	n.a.	Zhou et al. 2017 [10]
Pt 23	Fam. 11	c.1577C > T; c.1466 T > G	p.Ala526Val; p.Phe489Cys	n.a.	n.a.	Murray et al. 2017 [7]
Pt 24				n.a.	Normal (muscle)	
Pt 25	Fam. 12	c.1514G > A; c.1514G > A	p.Arg505His	Normal (plasma and CSF)	cI, I + III,II + III defects (muscle)	This report (Pt A); Orcesi et al. 2011 [13]
Pt 26	Fam. 13	c.1124A > G; c.381C > G	p.Tyr375Cys; p.Phe127Leu	Elevated (plasma), normal (CSF)	Normal (fibroblasts)	This report (Pt B)
Pt 27	Fam. 14	c.815 T > G; c.1043G > A	p.Phe272Cys; p.Arg348His	Elevated (plasma), normal (CSF)	Normal (fibroblasts)	This report (Pt C)

KARS impairment was first linked to peripheral neuropathy [8] in one patient (Pt 1) presenting with Charcot-Marie-Tooth neuropathy, developmental delay, self-abusive behavior, dysmorphic features, and vestibular Schwannoma. Compound heterozygous p.Leu133His and p.Tyr173SerfsX7 variants were identified. Functional analyses revealed that these two mutations severely affect enzyme activity.

Autosomal recessive nonsyndromic hearing loss was the second phenotype reported [9]. In affected individuals homozygosity for missense mutations (p.Asp377Asn or p.Tyr173His) in *KARS* was identified. (Pts 2–14/Fam. 2–4). Both variants were predicted to be damaging by multiple bioinformatics tools. The first case of *KARS* mutations associated with suspected mitochondrial disease was reported in 2013 [4]. The authors analyzed by exome sequencing a series of 102 patients with clinical and biochemical findings suggestive for mitochondrial disorders and identified compound heterozygous *KARS* mutations (p.Thr587Met; p.Pro228Leu) in a patient affected by psychomotor delay, hearing loss, ophthalmoplegia, dystonia and elevated CSF lactate level (Pt 15/Fam. 5). MRC activity on tissue was not investigated. No experimental proof was reported but, given the predicted severity of the mutations at highly conserved residues, the authors concluded that the observed mutations were likely the genetic cause of patient’s phenotype.

A more severe phenotype was reported in two 2 siblings (Pts 16–17/Fam. 6) with early onset visual impairment, progressive microcephaly, developmental delay, seizures and very subtle deep white matter loss on MRI [5]. The patients harbored compound heterozygous mutations (p.Arg466Trp; p.Glu553Lys) within a highly conserved region of the catalytic domain. A similar clinical presentation was reported in a patient who harbored a p.Ala57Pro missense change and a 7601-base pair deletion, encompassing the first three exons of the mitochondrial isoform of *KARS* (Pt 18/Fam. 7) [6]. Cardiac involvement associated with a deficiency of MRC complexes I and IV has been reported in two patients (Pts 19–20/Fam. 8–9), who carried novel biallelic *KARS* mutations [11, 12]. The first one presented a childhood-onset hypertrophic cardiomyopathy associated with seizures, developmental delay, in a patient harboring compound heterozygous p.Val476Asp and p.Ile346Thr mutations [11] while hypertrophic cardiomyopathy was the clinical hallmark in the second, a 14 years old patient with mild myopathic signs and cognitive disability (in spite of normal brain MRI) associated with p.Leu378His and p.Pro418Arg [12]. In both patients lactic acidosis was detected. In the first case, the mitochondrial enzyme defects were rescued by cDNA complementation with mitochondrial KARS, but not cytosolic form [11]. More recently, two mutations (p.Arg505His; p.Pro533Ser) have been reported in two siblings affected by early onset hearing loss, progressive cognitive impairment and psychiatric symptoms with onset in adulthood associated with leukoencephalopathy: brain MRI showed symmetrical confluent abnormalities in the frontal, periventricular white matter and in the corpus callosum [10]. Functional studies showed that both mutations decreased tRNA aminoacylation while p.Arg505His changed the secondary structure of KARS, leading to protein aggregation. Finally, *KARS* mutations (p.Ala526Val; p.Phe489Cys) were reported in two sisters affected by developmental delay, microcephaly, seizures, and sensorineural hearing loss; calcifications of left occipitoparietal junction were reported in one case (Pt 23). MRC enzymes activity in muscle biopsy was normal, lactate level was not available (Pt 24) [7].

In the present manuscript we reported about a new severe phenotype associated with biallelic *KARS* mutations. Our patients presented an early onset and progressive encephalopathy characterized by acquired microcephaly, developmental delay, spastic tetraparesis, epilepsy, sensorineural hypoacusia, visual impairment, microcytic hypochromic anaemia and failure to thrive. In addition patients B and C presented liver involvement. To our knowledge, this is the first report of hepatic involvement in this disease but the exact meaning of the reported anomalies has still to be understood. Nevertheless, in patient A no evidence of liver dysfunction was reported and liver ultrasound was normal.

In patient B, the severity of the phenotype, the clinical onset related to febrile illness and the presence of lactic acidosis suggested a mitochondrial disease that was directly investigated in spite of normal MRC and PDH activities in fibroblasts.

In patient C, clinical picture and lactic acidosis claimed the idea of a mitochondrial disorder as already suggested also in patient A, supported by mild lactate elevation at spectroscopy study. In *KARS*-mutant cases reported to date, mitochondrial disease was suspected and confirmed by biochemical diagnosis only in patients with cardiomyopathy (Pt 19 and Pt 20); elevated lactate level in CSF was detected in Pt 15 but biochemical studies on tissue were not performed. In other cases, metabolic analyses for mitochondrial disease were unremarkable or not performed. In 5 of 26 KARS patients reported to date, including our cases, both lactate level and biochemical studies (in different tissues) were performed and only in patients with cardiac involvement elevated lactate level corresponded to reduced MRC activity. Nevertheless the lack and heterogeneity of laboratory data does not permitted this phenotypic variability explanation. The MRI findings were similar in all three patients and characterized by progressive diffuse leukoencephalopathy and calcifications extending in cerebral, brainstem and cerebellar WM, with spinal cord involvement. Specifically, at the early stages of the disease the signal abnormalities were observed in the deep cerebellar WM and in the centrum semiovale. Progressively, an extensive diffuse WM involvement, including U fibers, posterior arm of the internal capsules, external capsules, thalami, cerebellar peduncles and brainstem, with selective bilateral symmetric involvement of the bulbar pyramids and lateral bulbar regions resembling the pattern of mitochondrial diseases were observed. The supratentorial WM involvement was characterized by uniform slight hyperintensity on T2WI, interrupted by marked foci of hypointensities due to calcifications. This appearance seems to be due to a demyelinating process an assumption supported also by the evidence of demyelination of the proximal intracisternal portion of the V cranial nerves (Fig. 1g).

Cerebral calcifications have a distinct pattern with initial involvement of deep cerebellar and cerebral periventricular WM and progressive extension to the thalami and internal capsules, in which a peculiar “boomerang appearance” was present. Calcifications were evident even in the initial phases of the disease and might not be a dystrophic epiphenomenon and so a secondary and aspecific event, but an intrinsic feature of the disorder. In the spine they were present in all 3 patients, even if with different severity, and were characterized by a peculiar bilateral and symmetrical distribution in the anterior horns, both extensively (Patient A) or spot-like (Patients B and C). On MRI, bilateral abnormal signal intensity in the lateral columns was also associated.

Patient B dysplayed a more severe cerebral atrophy and grey matter involvement (basal ganglia and cortex) but he underwent the first MRI later in life compared to the other two. As disease progressed, the radiological picture evolved toward a progressive cerebral atrophy in 2 patients (A and B).

WM involvement has been previously reported in few *KARS*-mutant patients but with a less severe pattern and restricted to supratentorial regions (Pt 16, Pt 17, Pt 21, Pt 22). It is interesting to note that the presence of cerebral WM and spinal cord signal abnormalities is a pretty rare association of neuroradiological features and it is typically observed in other aaRSs deficiencies, notably in *DARS* and *DARS2* related leukodystrophies [27, 28]. It is also a quite common finding in Iron-sulfur cluster related leukoencephalopathies, particularly those caused by *GLRX5* [29]*, ISCA2* [30], or *IBA57* [31] mutations. The association of cerebral WM abnormalities with spinal cord involvement should prompt to consider aaRSs-related diseases and particularly *KARS* mutations when calcifications are observed.

The extremely heterogeneous clinical presentation associated with *KARS* mutations is peculiar in the field of *aaRS*-related diseases which are usually characterized by strict genotype-phenotype associations, although a definite explanation of the molecular mechanisms underpinning this observation is still missing. Few examples of different phenotypes caused by mutations in the same *aaRS* gene have been reported (e.g. *AARS2* associated with either cardiomyopathy or leukoencephalopathy and ovarian failure [22, 32]). Differences in mode of inheritance and type of mutation cannot easily explain the variable clinical presentations since all the reported cases showed an autosomal recessive transmission of missense mutations. Only the patient described by Joshi et al. (Pt 18/Fam. 7) carried a large deletion, acting as a null allele, together with a missense mutation which disrupts the mitochondrial targeting signal, thus potentially affecting solely the mitochondrial isoform of *KARS*. All the other patients, irrespective of any evidence of mitochondrial dysfunction, harbored *KARS* variants which are predicted to strike both mitochondrial and cytosolic *KARS* isoforms. An effect of the affected functional domains was initially suggested, since the first mutations responsible for the neuropathic phenotype hit the anticodon domain whereas hearing loss associated mutations could be in the catalytic domain. However this hypothesis was not confirmed in the following reports and in the present review of all the *KARS* mutant patients. For instance, the mutations found in our patients, with an overlapping phenotype, are scattered throughout the gene (from amino acid 127 to 505) and affect either the anticodon-binding or the catalytic domain. The few functional studies which have been performed indicated that various functions/properties of KARS (e.g. tRNA aminoacylation, secondary structure) may be affected by different mutations. Nevertheless, no genotype/phenotype correlation was evident, even considering the residual enzymatic activity of the different mutant forms. Nevertheless a mutation specific effect cannot be excluded, since all the identified *KARS* mutations were reported in single cases/families; for instance, the cardiac phenotype in *AARS2* mutant patients seems to be strictly linked to the presence of a specific amino acid change. The only *KARS* mutation presents in two unrelated families was the p.Arg505His, identified in homozygosity in patient A and in compound heterozygosity with p.Pro533Ser in Pts 21–22; all these three individuals were characterized by leukodystrophy and hearing problems but the MRI features were not identical and other clinical symptoms were different (e.g. visual impairment and spastic tetraparesis were observed in patient A but not in the two siblings). Obviously, the partially different genotype may account for the phenotype diversities.

## Conclusion

With our report we define the molecular basis of the previously described Leukoencephalopathy with Brainstem and Spinal cord Calcification, that we propose to call LBSC similarly to *DARS* and *DARS2* related leukodystrophies, widening the spectrum of *KARS* related disorders, particularly in childhood onset disease suggestive for mitochondrial impairment. The review of previous cases does not suggest a strict and univocal genotype/phenotype correlation for this highly heterogeneous entity.

Moreover, our cases confirm the usefulness of search for common brain and spine MR imaging pattern and of broad genetic screening, in syndromes clinically resembling mitochondrial disorders in spite of normal biochemical assay.

## Additional files

Additional file 1: List of the genes present in the custom panels. (XLSX 12 kb)

Additional file 2: List of the candidate variants identified by WES in Patient A. (XLSX 10 kb)

Additional file 3: Information about the identified KARS mutations. (XLSX 8 kb)

## Abbreviations

aaRSs: Aminoacyl–tRNA synthetases
MRC: Mitochondrial respiratory chain
NGS: Next generation sequencing
PDH: Pyruvate dehydrogenase
tRNA: Transfer ribonucleic acid
WM: White matter
